# Evaluation of socket healing in patients undergoing bisphosphonate therapy: Experience of a single Institution

**DOI:** 10.4317/medoral.18787

**Published:** 2013-03-25

**Authors:** Gabriel F. kato, Rodrigo N. Lopes, Graziella C. Jaguar, Ana P. Silva, Fabio A. Alves

**Affiliations:** 1 DDS, DDS MSc, DDS PhD, DDS, DDS PhD. Stomatology Department, Hospital A.C. Camargo, São Paulo, Brazil; 2 DDS, DDS PhD. Stomatology Department, University of São Paulo, São Paulo, Brazil

## Abstract

Objective: To assess the clinical features of exodontias performed in cancer patients who have been receiving intravenous bisphosphonates (BPs). Study Design: This is a retrospective cohort study using a sample of 20 patients receiving BPs who had 62 teeth extracted. An univariate analysis was applied to calculate socket healing time (HT), comparing among exodontias performed according to cause, such as periodontal disease or caries, type of BP, and use of corticosteroid. In order to analyze the influence of each variable on HT, multiple statistical analyses were performed through logistic multiple regression. Results: From the 62 tooth extractions performed, 5 exodontias had evolved to 4 sites of bisphosphonate-related osteonecrosis of the jaws (BOJ). Of another 57 exodontias without development of BOJ, HT was significantly better for tooth extraction performed in patients receiving corticosteroid (p= .01), for tooth extracted due to caries (p= .04), and for extractions under pamidronate (p= .03). Sockets after exodontias due to periodontal diseases had OR= 5.22 (95% CI 1.73-133.66, p=0.01) for delayed HT, exodontias performed under corticosteroid use had OR=0.04 (95% CI 0.01-0.40, p<0.001), and exodontias performed under zoledronate had OR=0.31 (95% CI 0.08-1.25, p=0.10). Conclusions: Exodontias performed in patients under BP therapy had a low rate of BOJ occurrence. Zoledronate and periodontal diseases influence delayed socket healing. Adjuvant antibiotics could be relevant procedures aimed at reducing the risk of BOJ development.

** Key words:**Bisphosphonate; tooth extraction; osteonecrosis; jaw osteonecrosis; bisphosphonate-related osteonecrosis; socket healing.

## Introduction

Bisphosphonates (BPs) are compounds characterized by two carbon-phosphate bonds, which act on decreasing bone resorption, varying greatly from different classes of BPs. According to the presence of nitrogen bound to the chemical structure, BPs are classified as non-nitrogen BPs, such as etidronate, clodronate, and nitrogen BPs, such as risedronate, aledronate, pamidronate, and zoledronate (1). These drugs are widely used in the treatment of bone metabolism diseases, notably to reduce skeletally related events in patients with metastatic cancer, multiple myloma, Paget’s disease, and osteoporosis (2-4).

Adverse effects related to intravenous BPs generally include acute systemic inflammatory reaction, ocular inflammation, renal failure, nephrotic syndrome, and osteonecrosis of the jaws (5). Bisphosphonate-related osteonecrosis of the jaws (BOJ) is defined as a side effect to the inhibition of osteoclasts in which exposed and necrotic bone persisting for more than 8 weeks occurs in the maxillofacial region, which could be related to current or previous treatment with BPs, with no history of radiotherapy to the head and neck area (6). Although bone remodeling suppression related to BPs is well established, the entire mechanism of BOJ pathogenesis remains unclear (7).

Several studies have reported tooth extraction as a potential risk factor for the development of BOJ (6,8-10). The incidence of BOJ associated with exodontias varies between 36.7% and 73% of reported cases (11-13). The typical presentation is a nonhealing extraction socket or exposed jawbone with progression to sequestrum formation associated with localized swelling and purulent discharge. A couple of prospective cohort studies have established a specific preventive protocol for tooth extraction in patients using BPs with an expressive reduction of its occurrence, ranging between 0 and 2.7% (14-18).

To the best of our knowledge, no study has assessed the socket healing time (HT) after exodontias have been performed in patients under bisphosphonate therapy. Thus, the aim of the current study was to evaluate socket healing after exodontias in these patients.

## Material and Methods

-Study design and sample constitution

This research consisted of a retrospective observational cohort study in which exodontias performed in patients using BPs due to reduce the skeletally related events in patients with bone metastases were evaluated. A total of 20 patients met the criteria for inclusion. Sixty-two exodontias, performed between January 2004 and December 2010 at the Stomatology Department of Hospital A.C. Camargo, São Paulo, Brazil. The study was approved by the ethical committee of the institution and registered under protocol 1512/2011.

-Research criteria

Inclusion criteria consisted of patients who submitted to tooth extraction and who had received at least one application of intravenous BP.

Exclusion criteria consisted of patients who underwent radiotherapy in the region of head and neck. Futhermore, cases of tooth extractions performed in consequence of previous osteonecrosis related to BPs were also excluded.

-Data collected

The patient’s information was obtained from medical charts. Data were collected on gender, age, underlying disease, use of corticosteroid and duration of its administration, type of BP and doses administered, date of last BP infusion, antibiotic administration, teeth extracted, date and motive of exodontia, as well as date of alveolar socket healing.

Alveolar socket evaluation

The socket status after exodontias was classified into two forms:

- Healed socket: alveolar socket had been totally recovered by the mucosa and there was no osseous exposed or suppuration.

- Non-healed socket: during the follow-up period, the socket had no sign of healing. Consequently, bone exposure or local infection (pus drainage) could be observed.

**Figure 1:**
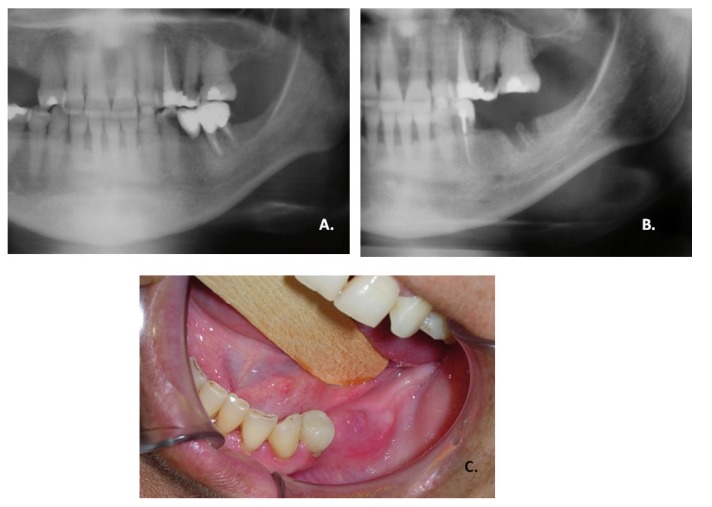
Non-healed socket after exodontia. A. Panoramic radiograph before exodontia. B. Panoramic radiograph after 3 months of exodontia; image suggests bone remodeling did not occurred in alveolar socket. C. Clinical aspect of alveolar socket after exodontia without mucosa coverage.

All patients were seen once a week and the socket healing time (HT) was also assessed. The HT was established as the period between the exodontias and the complete healing of the socket. The clinical aspects for non- healed socket can be observed in figure 1. Furthermore, only the cases which had healed socket were included for analysis of the HT.

-Statistical Analysis

Bivariate analysis was performed using the Mann-Whitney U test to analyze the differences on HT according to the type of BPs (pamidronate or zoledronate), corticosteroid, and motive of exodontia. Time of BP interruption was established through the interval of days between the date of the last BP infusion to the date of exodontia. This interval was correlated with HT in order to evaluate the influence of its interruption on the alveolar socket healing, through the Person’s Correlation Test. In order to analyze the influence of each variable (cause of exodontia, type of BPs, corticosteroid use) on HT, risk estimation was established for overdelayed socket healing through multiple logistic regression.

## Results

-General Results 

The sample consisted of 20 patients who were submitted to 62 tooth extractions. The median age was 62.2 years, ranging from 43 to 83 years, with 80% of the patients being women. Most of the patients were using BPs due to bone metastases of breast cancer ( Table 1).

**Table 1:**
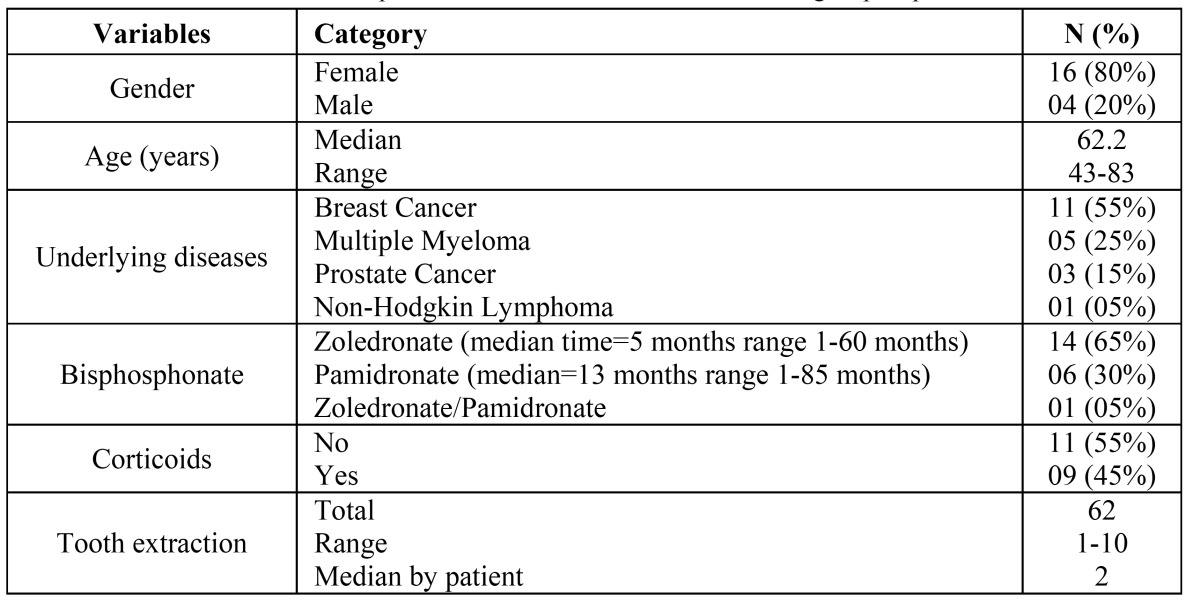
Clinical features of the 20 patients submitted to exodontias and using bisphosphonates.

A total of 62 teeth were extracted, 26 on the maxilla and 36 on the mandible. The median of dental extraction by patient was 2 teeth, ranging from 1 tooth to 10 teeth. Regarding BP type, 37 teeth were extracted in patients under zoledronate therapy and 25 under pamidronate. In addition, 29 teeth were extracted due to periodontal disease and 33 due to caries. For all exodontias, antibiotic adjuvant therapy was used, with 34 teeth extracted under amoxicillin, 22 amoxicillin and metronidazole, and 6 teeth extracted under clindamycin. Antibiotics were initiated one day prior to tooth extraction and the mean time of antibiotic therapy was 8.41+2.99 days.

A total of 34 exodontias were performed under corticosteroid use, 10 patients were receiving oral doses of dexamethasone which ranged from 20-40mg a day. The prescriptions had been performed due to the management of osseous pain related to bone metastases. The mean time of corticosteroid use was 11 months (median of 3 months, ranging from 1 month to 23 months).

-Bisphosphonate-related Osteonecrosis of the Jaws 

BOJ associated to tooth extraction was observed in 4 patients. Interestingly, all osteonecrosis sites were related to premolar extractions. One patient had been submitted to 2 exodontias in the same clinical session (teeth 44 and 45) and both teeth were extracted due to periodontal disease. The other three patients who developed BOJ had their teeth extracted due to carie (2 cases) and periodontal diseases (one case). With regard to BP type, 3 out of 4 patients were using zoledronate and the other patient was using pamidronate. The mean dose of BP was 18, ranging from 10 to 40 doses.

-Alveolar socket healing

Of the 16 patients without BOJ development, 11 were using zoledronate and 5 pamidronate (median of BP doses was 7, ranging from 1 to 85 doses).

**Figure 2:**
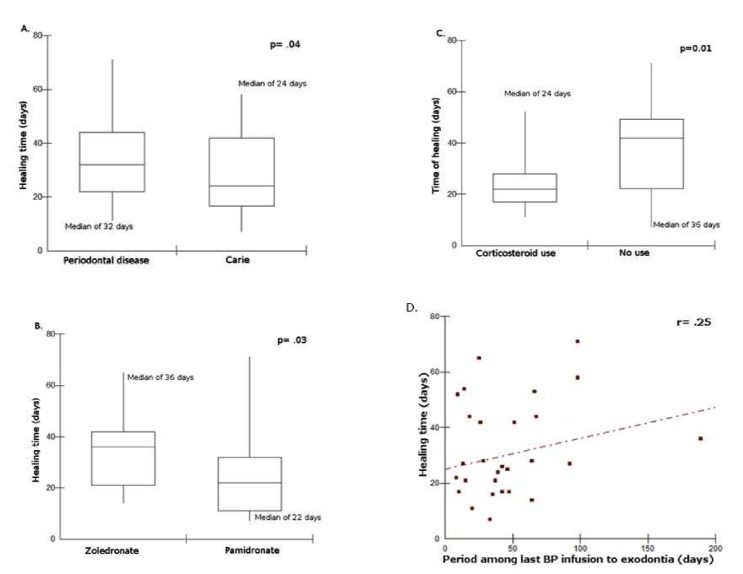
Univariate analysis of socket healing time (HT). Fig2a. Boxplot describing HT according to motive of exodontia. Fig2b. Boxplot describing HT according to type of BP. Fig2c. Boxplot describing HT according to corticosteroid use. Fig2d. Correlation between Socket healing time (HT) and period among last BP infusion to exodontia.

Of the 62 exodontias, 57 had healed alveolar socket at a median healing time of 27 days (mean 30.49+16.49, range 7-71 days). The exodontias performed due to caries had their sockets healed at a median HT of 24 days (mean 27.35±16.33, range 7 to 58 days), whereas the sockets related to exodontias due to periodontal disease healed at median HT of 32 days (mean 34.23±16.18, range 11-71 days) (Fig. 2).

Concerning the type of BP, exodontias in patients using pamidronate had a median HT of 24 days (mean 26.54±19.06, range 7 to 71 days), whereas in patients using zoledronate the median HT was 33 days (mean 33.36±13.93, range 14 to 65 days) (Fig. 2). Furthermore, patients receiving therapy with corticosteroid also had lower HT than non-corticosteroid patients, with a median HT of 22 days (mean 24.93±11.15 days) and 42 days (mean 36.25±19.16 days), respectively (Fig. 2). The mean time of the interval between the last BP dose to the date of the exodontia was 64.27 days (median of 36, ranging from 8 to 877 days). There was no correlation among this interval with HT (Fig. 2) (r=0.25; p=0.03).

According to the healing outcomes, 34 sockets were classified as expected socket healing in which HT ranged between 7 and 28 days, whereas 23 sockets were classified as delayed socket healing in which HT ranged between 36 and 71 days. Sockets after exodontias due to periodontal diseases presented OR=15.22 (95% CI 1.73-133.66, p=0.01) for delayed socket healing time compared with socket healing after exodontias due to caries, whereas sockets after exodontias performed under corticosteroid use had OR=0.04 (95% CI 0.01-0.40, p<0.001) compared with sockets with delayed healing after exodontias not under corticosteroid use. Furthermore, socket healing after exodontias performed under zoledronate had OR=0.31 (95% CI 0.08-1.25, p=0.10) compared with socket healing after exodontias performed under pamidronate ( Table 2).

**Table 2:**
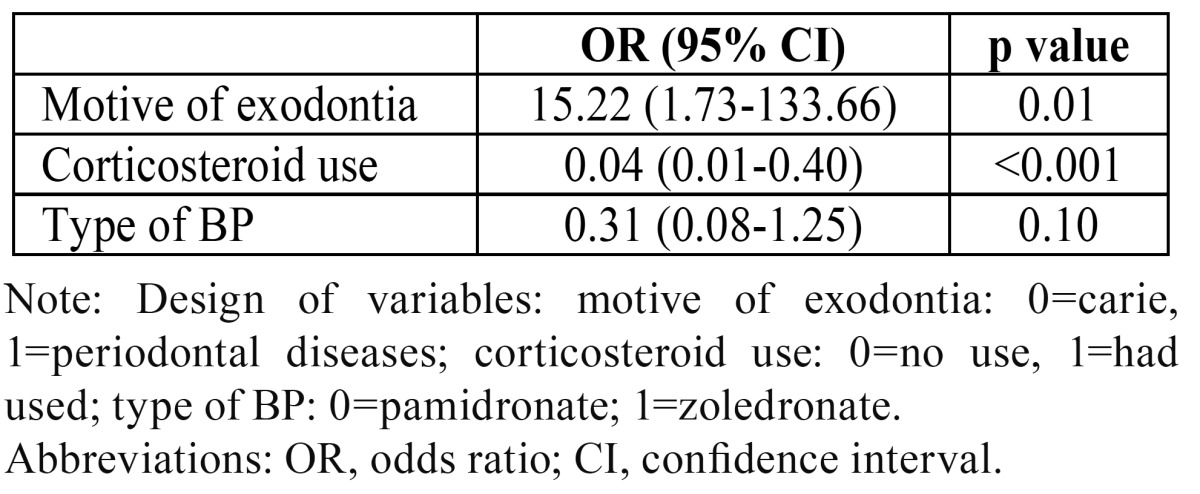
Estimative for occurrence of delayed socket healing time according to the variables analyzed through logistic regression model.

## Discussion

Suppression of bone turnover may be part of the pathophysiology of BOJ development (7,19). The basic premise of this hypothesis is that jaw bones have a high remodeling rate compared with other bones. However, BPs can diminish or inhibit bone turnover according to its potency and mechanism of action. Non-nitrogen BPs inhibit bone resorption by incorporation into cytotoxic adenosine triphosphate (ATP) analogues, whereas the mechanism of action of nitrogen BPs is related to its interactions with reactions such as mevalonate pathway, interfering with post-translational prenylation of proteins by a decrease of the formation of isoprenoid lipids such as farnesyl pyrophosphate and geranylgeranyl pyrophosphate, which affect cellular activity such as apoptosis in several cell types, including osteoclasts (1,2,20). Differences in potencies and binding affinities among BPs are known to affect the degree of remodeling suppression, as well as the dose and duration of BP therapy (7,21). Furthermore, zoledronate is considered the most potent BP. In the present study, 6 out of 20 patients were using pamidronate, and these patients had been submitted to 25 exodontias with one socket developing BOJ. Additionally, the other 13 patients were using zoledronate and were submitted to 36 exodontias, in which 3 had evolved to BOJ. A single patient was firstly treated by zoledronate and substituted by pamidronate due to kidney failure. This patient was submitted to one exodontia without BOJ development.

Bagan et al. (2009) pointed that BOJ is more related to previous dental extractions than to its spontaneous occurrence (22). Epithelialization is an essential step in post-intervention wound healing and it has been suggested that high doses of BPs have direct toxic effects on the oral epithelium. In addition, BPs can also inhibit normal healing of soft tissue lesions caused by either dental intervention or some other trauma, which would result in exposure of the bone prior to necrosis due to the release of BP from the adjacent injured bone (7,19). The present study criteria considered the term ‘socket healing’ as the socket totally covered by the mucosa, independently of bone formation. Besides the 57 sockets which had complete healing, the mean and median of HT were 30 and 27 days, respectively. Exodontias in patients under zoledronate had healed at a median of 33 days, whereas sockets under pamidronate had healed at median HT of 24 days. This difference was statistically significant through univariate analysis. Consequently, our results suggest that zoledronate interferes more in the socket healing process than pamidronate. Moreover, the mean time of the zoledronate use was lower than pamidronate. The influence of zoledronate in bone turnover was evaluated in beagle-dogs (21). The authors showed that the use of zoledronate for 6 months had produced nearly complete suppression (99%) on bone remodeling of the mandible. This scientific evidence highlighted that zoledronate interferes with bone remodeling and may also explain the delayed socket healing after exodontias were performed in patients under zoledronate therapy.

It is known that the BP nucleus carries a strong negative charge as a result of the clustering of oxygen atoms in the two phosphate groups, and thus, binds tightly to the positively charged surface of hydroxyapatite. As a consequence, incorporation into the bone mineral remains in the bone over a period of years (23). In the present study, the influence of BP interruption was calculated according to Pearson’s correlation test and there was no relation to the HT (r=.25). However, Saia et al. developed a preventive protocol for tooth extraction in patients under a duration time of 3 years on nitrogen-BPs therapy (16). The authors recommend BP interruption for 1 month after exodontias. In fact, there is no scientific evidence that BP interruption could promote better socket healing and more studies are necessary regarding this issue.

We reviewed the literature and found 5 other studies assessing exodontias in patients under BP therapy ( Table 3) (14-18). BOJ incidence ranged from 0 to 8.0%. In general, most of these studies recommend antibiotics as adjuvant therapy to perform the exodontia (15-18). Antibiotic adjuvant may be an important procedure to reduce the bacterial colonization of oral cavity and may also prevent BOJ occurrence as well as opportunistic bacterial infections at sockets after exodontias. Differently, Regev et al. (14) standardized tooth extraction with orthodontic elastics. The tooth was exfoliated in order to minimize inherent trauma related to exodontias; antibiotic prescription as adjuvant therapy was not mentioned.

**Table 3:**
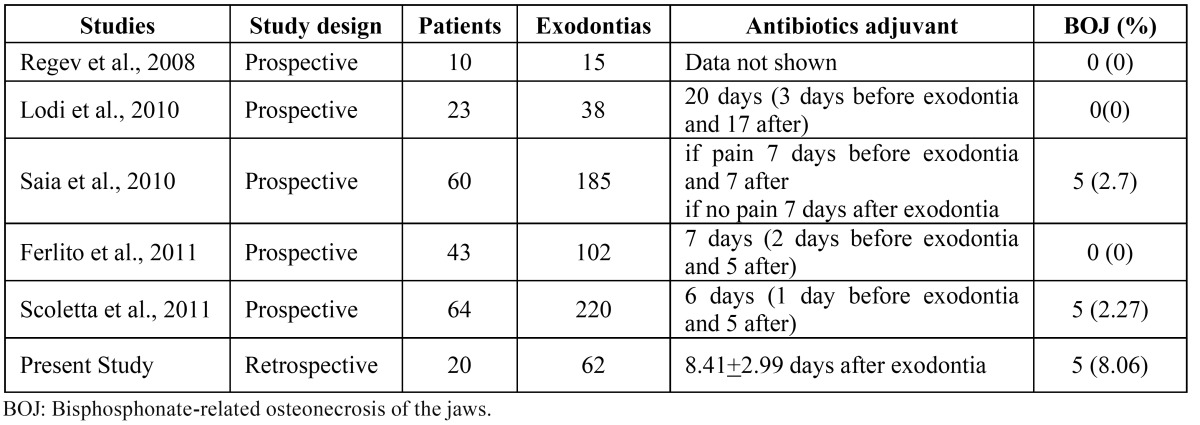
Literature review of studies that evaluated exodontias in patients using bisphosphonates.

An interesting data found in the present study was a difference between HT in exodontias performed due to caries (median HT of 24 days) and periodontal diseases (median HT of 32 days) (p=.04). Multiple analysis also confirmed that periodontal diseases increase HT of sockets after exodontias (OR 15.22; 95% CI 1.73-133.66, p=0.01). Therefore, exodontias due to periodontal diseases needed a major time to heal and, consequently, the socket would be more susceptible to infection due to oral pathogens.

For the present casuistic, exodontias were performed due to advanced periodontal diseases without possibilities of conservative treatment or unrestorable teeth related to caries. Periodontal diseases can be defined as complex bacteria-induced infections characterized by an inflammatory host response to plaque microbiota and their products. These microorganisms have virulence factors capable of causing massive tissue destruction both directly, through tissue invasion and the production of harmful substances, or indirectly, by activation of host defense mechanisms, creating an inflammatory infiltrate with potent catabolic activity that can interfere with normal host defense mechanisms. Then, this manifestation depends on an interaction between environmental, microbiologic agent and host-related factors (24). On the other hand, dental caries are characterized by the focal destruction of dental mineralized structures such as enamel and dentin due to colonization by acidogenic and acidophilic bacteria. Additionally, the degree and severity of the infection presented prior to exodontia are higher for teeth extracted due to periodontal diseases. Periodontal diseases could be characterized by intense tissue destruction due to inflammatory response, as well as bacteria production of harmful substances. At the same time, it is noted that exodontias, due to periodontal diseases, reported higher HT and is described as a factor for delayed HT.

Exodontias under corticosteroid use is also observed to have presented lower HT confirmed by bivariate analysis, and reduced HT according to a multiple analysis. Despite the fact that corticosteroid use for a longer period promotes an immunological side effect, it could not be denied that corticosteroids are potent anti-inflammatory drugs with good affinity at oral cavity tissues. Dental extractions are surgical procedures that involve inherent trauma caused by the technical manipulation of the oral soft tissue, the remaining tooth and the alveolar socket bone. All the teeth were extracted with the most adequate techniques for an atraumatic tooth extraction using mainly elevators and forceps (if necessary). However, inherent trauma involves an inflammatory response that probably was reduced for patients using corticosteroid due to medical prescription. Paradoxically, the fact that corticosteroid use could improve HT after exodontias under BPs, all 4 cases of BOJ occurrence after exodontias were performed under corticosteroid use at a mean time of 6.2 months (range 1 to 16 months). Adjuvant anti-inflammatory drugs may be an important procedure to reduce inflammation due to inherent trauma of tooth extraction, as well as due to the proper pathological process that makes tooth extraction necessary.

In conclusion, exodontias performed in patients under BP therapy had a low rate of BOJ occurrence. Indeed, socket healing after exodontias happened in delayed healing time, mostly, in exodontias due to periodontal diseases and under zoledronate use. Therefore, the prescription of adjuvant antibiotics and modulators of inflammation could be important procedures aiming to reduce the healing time of exodontias performed in patients under BP. Moreover, from our viewpoint, exodontias, when necessary, must be performed by expert dentists and follow-up must occur until the complete covering of the socket by mucosa.

## References

[1] Fleisch H (2002). Development of bisphosphonates. Breast Cancer Res.

[2] Devogelaer JP (2000). Treatment of bone diseases with bisphosphonates, excluding osteoporosis. Curr Opin Rheumatol.

[3] Dalle Carbonare L, Zanatta M, Gasparetto A, Valenti MT (2010). Safety and tolerability of zoledronic acid and other bisphosphonates in osteoporosis management. Drug Healthc Patient Saf.

[4] Coleman RE, McCloskey EV (2011). Bisphosphonates in oncology. Bone.

[5] Tanvetyanon T, Stiff PJ (2006). Management of the adverse effects associated with intravenous bisphosphonates. Ann Oncol.

[6] Ruggiero SL, Dodson TB, Assael LA, Landesberg R, Marx RE, Mehrotra B (2009). American Association of Oral and Maxillofacial Surgeons position paper on bisphosphonate-related osteonecrosis of the jaws. J Oral Maxillofac Surg.

[7] Allen MR, Burr DB (2009). The pathogenesis of bisphosphonate-related osteonecrosis of the jaw: so many hypotheses, so few data. J Oral Maxillofac Surg.

[8] Yoneda T, Hagino H, Sugimoto T, Ohta H, Takahashi S, Soen S (2010). Bisphosphonate-related osteonecrosis of the jaw: position paper from the Allied Task Force Committee of Japanese Society for Bone and Mineral Research, Japan Osteoporosis Society, Japanese Society of Periodontology, Japanese Society for Oral and Maxillofacial Radiology, and Japanese Society of Oral and Maxillofacial Surgeons. J Bone Miner Metab.

[9] Urade M, Tanaka N, Furusawa K, Shimada J, Shibata T, Kirita T (2011). Nationwide Survey for Bisphosphonate-Related Osteonecrosis of the Jaws in Japan. J Oral Maxillofac Surg.

[10] Bocanegra-Pérez MS, Vicente-Barrero M, Sosa-Henríquez M, Rodríguez-Bocanegra E, Limi-ana-Ca-al JM, López-Márquez A (2012). Bone metabolism and clinical study of 44 patients with bisphosphonate-related osteonecrosis of the jaws. Med Oral Patol Oral Cir Bucal.

[11] Marx RE, Cillo JE, Ulloa JJ (2007). Oral bisphosphonate-induced osteonecrosis: risk factors, prediction of risk using serum CTX testing, prevention, and treatment. J Oral Maxillofac Surg.

[12] Bedogni A, Saia G, Bettini G, Tronchet A, Totola A, Bedogni G (2011). Long-term outcomes of surgical resection of the jaws in cancer patients with bisphosphonate-related osteonecrosis. Oral Oncol.

[13] Junquera L, Gallego L, Cuesta P, Pelaz A de Vicente JC (2009). Clinical experiences with bisphosphonate-associated osteonecrosis of the jaws: analysis of 21 cases. Am J Otolaryngol.

[14] Regev E, Lustmann J, Nashef R (2008). Atraumatic teeth extraction in bisphosphonate-treated patients. J Oral Maxillofac Surg.

[15] Lodi G, Sardella A, Salis A, Federica D, Tarozzi M, Carrassi A (2010). Tooth extraction in patients taking intravenous bisphosphonates: a preventive protocol and case series. J Oral Maxillofac Surg.

[16] Saia G, Blandamura S, Bettini G, Tronchet A, Totola A, Bedogni G (2010). Occurrence of Bisphosphonate-Related Osteonecrosis of the Jaw After Surgical Tooth Extraction. J Oral Maxillofac Surg.

[17] Scoletta M, Arduino P, Pol R, Arata V, Silvestri S, Chiecchio A (2011). Initial Experience on the outcome of teeth extractions in intravenous bisphosphonate-treated patients: a cautionary report. J Oral Maxillofac Surg.

[18] Ferlito S, Puzzo S, Liardo C (2011). Preventive protocol for tooth extractions in patients treated with zoledronate: a case series. J Oral Maxillofac Surg.

[19] Reid IR, Bolland MJ, Grey AB (2007). Is bisphosphonate-associated osteonecrosis of the jaw caused by soft tissue toxicity?. Bone.

[20] Roelofs AJ, Thompson K, Gordon S, Rogers MJ (2008). Molecular mechanisms of action of bisphosphonates: current status. Clin Cancer Res.

[21] Allen MR, Kubek DJ, Burr DB, Ruggiero SL, Chu TM (2011). Compromised osseous healing of dental extraction sites in zoledronic acid-treated dogs. Osteoporos Int.

[22] Bagan JV, Jiménez Y, Hernández S, Murillo J, Díaz JM, Poveda R (2009). Osteonecrosis of the jaws by intravenous bisphosphonates and osteoradionecrosis: a comparative study. Med Oral Patol Oral Cir Bucal.

[23] Cornish J, Bava U, Callon KE, Bai J, Naot D, Reid IR (2011). Bone-bound bisphosphonate inhibits growth of adjacent non-bone cells. Bone.

[24] Bascones-Martínez A, Muñoz-Corcuera M, Noronha S, Mota P, Bascones-Ilundain C, Campo-Trapero J (2009). Host defence mechanisms against bacterial aggression in periodontal disease: Basic mechanisms. Med Oral Patol Oral Cir Bucal.

